# Food Dosage

**Published:** 1916-09

**Authors:** 


### Food Dosage.

"For the ordinary adult, the amount of nitrogen required to replace daily waste, is contained in a piece of meat the size of an ordinary chop." This text is taken at random from an exchange, the original source we do not know. Some time ago, we took what we considered an ordinary lamb chop, carefully cut out the lean meat and weighed it—25 grams. Lean meat contains about 20% of proteid—5 grams for that chop. The older authorities state that the average adult requires about 100 grams of proteid a day; Chittenden, who has made exhaustive experiments to determine how low a proteid ration is possible, considers 50 grams the practical minimum.

250 grams, then, represents the amount of meat necessary to furnish the proteid ration, according to Chittenden; 500 grams according to the older authorities, supposing of course that no other source of proteid is eaten. A pound contains about 453 grams. A chop containing more than a pound or even more than half a pound of lean meat is not exactly ordinary, although in England, full grown mutton is used more than in America and the gross weight of a chop may be half a pound.

Dietetics is an extremely important branch of therapeutics, it deals with ponderable remedies. By actual scientific and, at the same time, practical experiment, we know how much of the organic nutritive principles is required to furnish energy and repair waste and as definitely as for crude drugs, the percentage of organic principles in crude foods.

Suppose that we knew that opium, any of its Galenicals or morphine, produced certain physiologic effects but did not know either the dose of morphine, or the assayed strength of opium or its Galenicals in terms of the active principle, or the empiric dose of any of the officinal preparations. Suppose that we told a pain-afflicted patient to get some tincture of opium and to take some, not too much, but a reasonable quantity, or as much as he could swallow. Except that an overdose of proteid, fat or carbohydrate does not produce as conspicuous symptoms as an overdose of morphine and that most persons have a natural appetite that guides them somewhat in their choice of food, very similar therapeutic failures, in either direction will follow if we do not know approximately the percentage of nutriments in ordinary food stuffs, how much is needed, and do not follow at least an approximate dosage in ordering foods.

Many physicians can pour liquids and powders out of the vials of their medicine cases, and judge the dose quite accurately by the eye. How many can guess, even approximately, how much bread, butter, meat, etc., they or their patients eat?

Do you, for instance, realize, that a quart of milk a day represents less than a third of an average ration? Do you know that it would take 29 eggs to equal the day's ration? Did you realize that the quotation at the beginning of this column was incorrect, as quickly, for instance as if a patient had given you a one dollar bill for a ten? Do you think that a 5c package of crackers weighs a pound? If so, study food dosage. It is hard work but just as necessary as studying dosage and composition of Galenical drugs. Don't understand us to say that a person should drink three quarts of milk and more a day, or eat 29 eggs. If a man stated that his wages were 300 cents a day, it would not mean that he could conveniently receive his pay in 300 coppers.

Only a short time ago, we read of a boy, treated unsuccessfully for months, by his family physician for an obscure ailment, turned over to a specialist in vain, finally sent to live with a relative who was a physician and a man of common sense. And the whole trouble was that the child admired his father so much that he imitated his habits of eating and was suffering from an overdose of proteid, fat and carbohydrate. Some time ago, we made a faecal examination in a similar case—not realizing its nature— and found, no particular abnormality, not even indigestion in the strict sense but simply too much of everything; total faecal mass, muscle oblongs, fats, vegetable residue.

Food dosage is no recondite matter. Typhoid fever has an average mortality of nearly 10%. Is it the infection that kills or, possibly, five weeks of burning up of ingesta and tissue, on a diet of a quart of milk a day, less than 1/3 the standard ration; perhaps with part of the milk replaced with meat juice and extracts at about 1/3 the caloric value of milk, not to mention the lack of balance between the nutrients of the ingesta and the demands of the system for a proper proportionate distribution of proteid, fat and carbohydrate?

Years ago, we saw a case in which a most skillful gastrostomy had been done to save a patient from starving and, then he was fed (?) on about 200 c.c. of meat extract, containing about 5% of proteid and practically nothing else —10 grams of proteid, about 40 calories net, approximately 1/60 of the total daily requirement. And, very logically, the patient died. Tincture or roast, opium or beef, morphine or proteid, it is a matter of percentage and dosage. In the former the danger is more often from overdose, in the latter from underdose but, for both drug and food, we must strike the happy mean of safe efficiency.

